# Urine Albumin Excretion Is Associated with Cardiac Troponin T Detected with a Highly Sensitive Assay in a Community-Based Population

**DOI:** 10.1371/journal.pone.0135747

**Published:** 2015-08-24

**Authors:** Wenkai Xiao, Ping Ye, Ruihua Cao, Xu Yang, Yongyi Bai, Hongmei Wu

**Affiliations:** Department of Geriatric Cardiology, Chinese PLA General Hospital, No. 28, Fuxing Road, Beijing, 100853, China; Nagoya University, JAPAN

## Abstract

**Background:**

Urine albumin excretion is an important predictor of adverse cardiovascular events. Minimally elevated levels of serum cardiac troponin T (cTnT), a marker of cardiomyocyte micronecrosis, can be detected with high sensitivity cTnT (hs-cTnT) assays. The purpose of this study was to investigate the relationship between alterations in albuminuria and serum hs-cTnT levels in a community-based population.

**Methods:**

We examined the association between the urine albumin/creatinine ratio (UACR) and hs-cTnT levels in 1354 participants without overt cardiovascular disease in a community-based, cross-sectional study in Beijing, China.

**Results:**

With the highly sensitive assay, cTnT levels were detectable in 90.5% of our subjects. The median (interquartile range) concentrations of hs-cTnT were 7 (5–10) pg/mL. After adjustment for several factors, UACR (odds ratio: 1.40; 95% confidence interval: 1.08–1.65; P = 0.002) was associated with a higher likelihood of elevated hs-cTnT (≥14 pg/ mL), whereas the relationship between UACR and a higher presence of detectable hs-cTnT (≥ 3 pg/ mL) was not significant. In addition, a fully adjusted logistic regression analysis revealed that compared with participants in the lowest UACR quartile, those in the highest quartile had a 2.43- fold (95% CI: 1.25–5.08; P = 0.006) increased risk of elevated hs-cTnT.

**Conclusions:**

Higher urine albumin excretion is associated with elevated hs-cTnT among persons without clinically evident cardiovascular disease, suggesting that albuminuria may be a potential risk factor for subclinical cardiovascular disease in the general population.

## Introduction

A close pathophysiologic relationship between the kidney and heart is well known. Recently many studies have suggested that urinary albumin excretion (UAE) is strongly associated with an increased risk of cardiovascular disease (CVD) [1–2]. It was reported in the CHARM study that microalbuminuria or macroalbuminuria increased the mortality rate by 60–80% in chronic heart failure [3]. Also, the HOPE study indicated that every 0.4 mg/mmol (equivalent to 3.01mg/g) increment in urine albumin creatinine ratio (UACR) conferred a 5.9% increase of major cardiovascular events [4]. However, the mechanisms underlying the relationship are still unclear but are thought to reflect a widespread vascular endothelial damage [5–6], dysfunction of the coagulation and fibrinolytic systems [7] and overexpression of neurohumoral factors [8].

Cardiac troponin T (cTnT) is a sensitive and specific marker of ischemic myocardial damage and is a widely used predictor of cardiovascular events [9]. A highly sensitive assay for cTnT (hs-cTnT) has recently been developed. Because this highly sensitive assay detects much lower levels of myocardial injury than prior assays, it may be useful for studying the earliest stages of heart disease. Hs-cTnT independently predicts cardiac or non-cardiac mortality in populations with or without cardiovascular disease [10–11] and has been used as a marker to predict future cardiovascular events in the general population [12]. However, information on the association between levels of hs-cTnT and UAE is currently limited, only a few investigations have evaluated their association in patients with chronic kidney disease, and the results were controversial [13–14].

We hypothesized that hs-cTnT is elevated in subjects with higher UAE, not due to decreased renal clearance, but because this marker truly reflects subclinical cardiac damage. Therefore, the objective of this study was to test the hypothesis that increased UAE (quantified by UACR) would be independently associated with subclinical myocardial injury measured by hs-cTnT in a community-based cohort without clinically evident CVD.

## Subjects and Methods

### Study design and population

This was a community-based study of individuals living in the Pingguoyuan area of the Shijingshan district, a metropolitan area of Beijing, China. Originally, a total of 1447 participants (who were at least 45 years old) were recruited for a routine health check-up between March and September 2013, but 11 subjects had missing data. Eventually, after excluding 82 participants for overt cardiovascular disease (defined as self-reported myocardial infarction, percutaneous transluminal coronary angioplasty, coronary artery bypass graft, or cerebrovascular accident), 1354 participants were included. The study was approved by the ethics committee of the Chinese People’s Liberation Army (PLA) General Hospital, and each participant provided written informed consent.

### Clinical data collection

Information on demographics, clinical history, and lifestyle was obtained by self-report on standardized questionnaires administered at the visit. Physical examinations and interviews were carried out by trained medical doctors, as described in detail previously in published document [15].

### Biochemical measurements

After an overnight fast of 12 hours, blood samples were obtained from all subjects. Participants with no history of diabetes were given a standard 75-g oral glucose tolerance test (OGTT), whereas, for safety reasons, participants with self-reported history of diabetes were given a steamed bun that contained approximately 80 g of complex carbohydrates. Concentrations of serum creatinine, lipid, glucose, plasma homocysteine and uric acid were determined using Roche enzymatic assays. Concentrations of hs-cTnT were determined using an Elecsys Troponin T high sensitive assay, details of the measuring method have been published elsewhere [16]. The lower detection limit of the hs-cTnT assay was 3 pg/mL, which was used as the cut-off point in the present analysis. The 99th percentile for a healthy subpopulation has been reported to be 14 pg/mL [17], and hs-cTnT levels ≥14 pg/mL were considered elevated.

UAE was expressed as the UACR and was evaluated in a random morning spot urine specimen. Each subject’ urinary albumin concentration was measured using immunoturbidimetric method (Beijing Atom High-Tech, Beijing, China), and urinary creatinine concentration was measured with Jaffe’s kinetic method on an automatic analyzer (Hitachi, Tokyo, Japan). UACR was calculated by dividing the urinary albumin concentration by the urinary creatinine concentrations and expressed in milligrams per gram.

### Definition of variables

Albuminuria was defined as a UACR of ≥30 mg/g (microalbuminuria, 30 to 299 mg/g; macroalbuminuria, ≥300 mg/g). A UACR <30 mg/g was defined as normoalbuminuria. Renal function was evaluated by the estimated glomerular filtration rate (eGFR). eGFR was calculated using the Chinese modifying modification of diet in renal disease (C-MDRD) equation, details of the computing equation have been published elsewhere [16]. The definition of hypertension [18] and diabetes mellitus [19] were according to the standard diagnostic criteria.

### Statistical analysis

Study participants were divided into UACR quartiles for descriptive purposes. The hs-cTnT levels are presented as categorical variables, they were classified as undetectable, detectable or elevated. In addition, when UACR was entered as a continuous variable, the natural logarithmic (ln) transformation of UACR was used because the UACR distribution was skewed. Differences in baseline levels of clinical characteristics across UACR quartiles were analyzed by one-way analysis of variance (ANOVA) or Cuzik’s nonparametric trend test for continuous variables, and chi-squared tests were performed for categorical variables.

We assessed the associations of the UACR measurements with manifestations of subclinical myocardial damage (detectable hs-cTnT versus undetectable and elevated hs-cTnT versus non-elevated) using logistic regression models. Multivariate logistic regression analysis was performed to obtain the odds ratios (ORs) and 95% confidence intervals (CIs). We implemented 4 models for the covariates: unadjusted model and regression models were adjusted for age and sex (Model 1), with subsequent adjustment for traditional cardiovascular risk factors and eGFR (Model 2), and finally with additional adjustment for cardiovascular drugs use (Model 3). In addition, to better understand the association between different UACR quartiles and elevated hs-cTnT, multivariate logistic regression analysis was repeatedly performed, and the quartile 1 UACR value was used as the reference.

Statistical analyses were performed using the SPSS statistical package software (version 17.0; SPSS Inc., Chicago, IL, USA). A two-sided value of P < 0.05 was considered significant.

## Results

### Clinical characteristics of subjects categorized by UACR level

Altogether, we included 1354 participants in the present study. The mean age of participants was 65.8 ± 10.1 years, and 58.8% were female. The median UACR was 11.68 (quartiles 1 to 3: 7.24–22.40) mg/g. Micro- and macroalbuminuria were noted in 200 (14.8%) and 31 (2.3%) participants, respectively. Participants were divided into four groups based on UACR quartiles (<7.24, 7.24–11.68, 11.69–22.40, >22.40 mg/g). S1 Table shows the trends for clinical and laboratory characteristics across progressive UACR quartiles. Participants with elevated UACR were more likely to be older and had a higher rate of cardiovascular risk factors including hypertension, DM, and increased body mass index. Progressively higher UACR quartiles were significantly associated with higher systolic blood pressure and blood glucose and reduced eGFR.

### Concentration and distribution of hs-cTnT concentrations

With the use of the high sensitive assays, 1225 (90.5%) subjects had detectable hs-cTnT (≥3.0 pg/mL). The median value of detectable hs-cTnT concentrations was 7 pg/mL (quartiles 1 to 3: 5–10 pg/mL). A total of 138 (10.2%) subjects had hs-cTnT concentrations ≥14 pg/mL. In addition, gradual increases in serum hs-cTnT levels were observed with increasing UACR (S1 Table).

### The association between UACR and hs-cTnT

When hs-cTnT was considered as a dichotomous variable (detectable versus undetectable and elevated versus non-elevated), on univariate logistic regression analyses, UACR levels were significantly related to the presence of detectable and elevated hs-cTnT (P ≤ 0.05 for each). In the three adjusted models, the relationship between UACR and elevated hs-cTnT remained statistically significant, whereas the association with detectable hs-cTnT disappeared. The significances of variables in the adjusted models are presented in Table 1.

**Table 1:** Relationship between UACR and the presence of elevated hs-cTnT.

	Elevated hs-cTnT		
	Odds ratios	95% CI	P-value
Unadjusted	1.67	1.44–1.94	<0.001
Model 1	1.55	1.31–1.84	<0.001
Model 2	1.42	1.18–1.72	<0.001
Model 3	1.40	1.08–1.65	0.002

Data are presented as odds ratios (per SD increase in the natural logarithm of the urinary albumin to creatinine ratio level) and corresponding 95% confidence intervals (CIs). Hs-cTnT levels ≥14 pg/mL were considered as elevated hs-cTnT. Model 1 = adjusted for age and sex. Model 2 = model 1 plus hypertension; diabetes mellitus; current smoking; body mass index; systolic and diastolic blood pressures; and levels of plasma triglyceride, low- and high-density lipoprotein cholesterol, fasting blood glucose, 2-h postprandial blood glucose, homocysteine levels and estimated glomerular filtration rate. Model 3 = model 2 plus use of (used = 1; unused = 0) angiotensin-converting enzyme inhibitors, angiotensin-receptor blockers, β-blockers, antiplatelet drug, statins.

Every SD increase in ln UACR was associated with a 1.40-higher likelihood of the presence of elevated hs-cTnT (OR: 1.40; 95% CI: 1.08–1.65, P = 0.002; Model 3, Table 1).

### UACR and the risk of elevated hs-cTnT across quartiles

A multivariate logistic regression analysis was performed with the first UACR quartile as the reference. In the univariate model, only UACR quartile 4 was associated with elevated hs-cTnT. After adjusting for variables significantly associated with higher hs-cTnT levels or after further adjustment for eGFR, the association remained strong for elevated hs-cTnT risk (OR: 2.43; 95% CI: 1.25–5.08; P = 0.006) in quartile 4 of UACR (Model 3, Table 2). Subjects in quartiles 2 and 3 did not have an increased likelihood of elevated hs-cTnT.

**Table 2:** Relationship between UACR and elevated hs-cTnT risk across quartiles.

UACR (mg/g)	Q1	Q2	Q3	Q4
	<7.24	7.24–11.68	11.69–22.40	>22.40
Univariable model				
Odds ratio	1.00	1.337	1.615	4.205
95% CI	Reference	0.683–2.617	0.844–3.093	2.351–7.520
P		0.397	0.148	<0.001
Multivariable model 1				
Odds ratio	1.00	1.364	1.547	3.279
95% CI	Reference	0.667–2.788	0.773–3.096	1.743–6.169
P		0.395	0.218	<0.001
Multivariable model 2				
Odds ratio	1.00	1.268	1.329	2.615
95% CI	Reference	0.593–2.713	0.636–2.780	1.315–5.200
P		0.540	0.449	<0.001
Multivariable model 3				
Odds ratio	1.00	1.114	1.205	2.432
95% CI	Reference	0.571–2.534	0.615–2.577	1.254–5.083
P		0.612	0.480	0.006

The quartile 1 level of UACR was used as the reference. Regression models were adjusted for Models 1, 2 and 3 (the same as described in Table 1).

## Discussion

In a community-based study of subjects without clinically evident CVD, we demonstrated that UACR was independently associated with levels of hs-cTnT, a reliable indicator of subclinical myocardial injury and disease prognosis. In addition, higher UAE level was strongly associated with the presence of elevated hs-cTnT, even after adjustment for potential confounding factors. To our knowledge, the association between UAE and hs-cTnT has yet not been specifically studied in the general population, these data reinforce the utility of albuminuria as a biomarker in clinical practice.

Using conventional assays, the prevalence of detectable cTnT in the 30- to 65-year-old general population was approximately 0.7% [20]. In fact, unrecognized myocardial ischemia and even unrecognized myocardial injury were common [21]. Recent advances have resulted in the development of hs-cTnT assays with greater sensitivity and lower limits of detection than previous version [22], therefore more subjects with subclinical myocardial injury can be identified earlier [23]. In the present study, we found that circulating hs-cTnT levels were detectable in most subjects (90.5%) and approximately 11.0% of subjects had elevated hs-cTnT levels, a substantial proportion of subjects had detectable hs-cTnT compared with other community-based studies [12, 23–24]. The prevalence of detectable hs-cTnT varied markedly with age [12]. The subjects in the present analysis were older and had more cardiovascular risk factors than those in previous studies, which may help to explain the difference in hs-cTnT levels.

The interaction between the heart and kidney has been explored for a long time. One recent study have demonstrated that members of the general population with both elevated albuminuria and reduced eGFR were at the highest risk for adverse cardiovascular outcomes [1]. UAE is an important predictor of adverse cardiovascular events in various populations, and a positive correlation between the degree of albuminuria and event severity has been reported [25–27]. Prior investigations evaluating the association between albuminuria and hs-cTnT mainly focused on patients with chronic kidney disease, and the results were controversial [13–14, 28]. A cross-sectional study in the chronic renal insufficiency cohort [13] revealed that UACR did not show a linear association with hs-cTnT, only subjects with UACR ≥ 1000 mg/g had higher hs-cTnT compared to those with UACR <30 mg/g. We examined the association of UACR with hs-cTnT in participants without CVD in a community-based study. Although most UACRs (82.9%) were below the clinical threshold for microalbuminuria, an independent association was still present. The findings of the present study support the hypothesis that UAE is a predictor of subclinical myocardial injury independent of eGFR and other traditional cardiovascular risk factors.

The pathophysiologic mechanisms of the correlation between UAE and hs-cTnT are unclear. One potential mechanism is that these two entities share several common risk factors; low-grade albuminuria is considered to be related to inflammation, diabetes, and hypertension [29], which are the risk factors for heart disease and may produce damage both organs over time. Recently, the relation between remission/reduction in microalbuminuria and reduction in cardiovascular events has been reported in patients with diabetes and essential hypertension [30–31]. However, even after adjustment for these factors, our data showed that slightly elevated UACR level remained associated with elevated hs-cTnT. Alternatively, the association could be in part due to endothelial dysfunction, which occurs in the settings of both albuminuria and abnormal cardiac mechanics [32–33]. Albuminuria is considered to be the consequence of endothelial dysfunction and abnormal vascular permeability in the renal glomerulus [34–35]. Some results suggested that microvascular bed dysfunction in the heart is paralleled by the same phenomenon in the kidney, and myocardial microangiopathy could lead to scattered myocardial fibrosis and necrosis, which can be manifested by increased hs-cTnT release [36].

After we adjusted for demographic factors, the association between detectable hs-cTnT and UACR was lost. Hs-cTnT was detectable in the overwhelming majority of our subjects, and the small number of participants in the undetectable group could have reduced the statistical power of our results. Higher UAE, including levels below the clinical threshold for microalbuminuria, was independently associated with a higher frequency of elevated hs-cTnT. Early vascular and myocardial changes may be present when UACR is below the microalbuminuria cut-off [37–38]. In a community- based sample of middle-aged nonhypertensive and nondiabetic individuals, an increasing UACR that was well below the threshold of microalbuminuria predicted CVD development [39]. Thus, even UAE in the high normal range may prove useful as an early marker of target organ injury and potentially help identify patients who require more intensive medical intervention.

The present study has several limitations. First, because of the inherent problems associated with cross-sectional design, the present study cannot identify causal relationships. Accordingly, our observations need to be confirmed in large-scale prospective studies. Second, subjects with known cardiovascular disease were excluded based on history, not on cardiac imaging, some subjects with undetected coronary artery disease may have been included. Third, we evaluated UAE from a single morning urine sample rather than 24-h urine or multiple samples. Although UACR in single spot samples fluctuate, the use of spot samples for UACR is recommended and the results correlate well with those of 24-h and multiple samples [40–41].

## Conclusion

In this community-based study of persons without clinically evident CVD, there was a cross-sectional association between UAE and hs-cTnT levels. These findings suggest that low-grade albuminuria could be an early marker of subclinical cardiovascular disease in this population and help identify persons in need of a specific cardiovascular risk management.

## Supporting Information

S1 Table Clinical Characteristics by UACR Quartile. (DOC)
