# Supplementary material for: Urine Albumin Excretion Is Associated with Cardiac Troponin T Detected with a Highly Sensitive Assay in a Community-Based Population
Source: PLoS One. 2015 Aug 24;10(8):e0135747. doi: 10.1371/journal.pone.0135747 (PMC4547701; doi:10.1371/journal.pone.0135747)
Supplement: S1 Table — (DOC) [file pone.0135747.s001.doc]

**Supplementary Table 1. Clinical Characteristics by UACR Quartile**

| Clinical characteristics | Q1 |  | Q2 |  | Q3 |  | Q4 |  | P Value |
| --- | --- | --- | --- | --- | --- | --- | --- | --- | --- |
|  | n=339 |  | n=338 |  | n=338 |  | n=339 |  |  |
| UACR range (mg/g) | <7.24 | | 7.24-11.68 | | 11.69-22.40 | | >22.40 | |  |
| Age (years) | 63.28±10.65 | | 64.63±10.52 | | 65.86±11.31 | | 69.36±10.89 | | <0.001 |
| Male sex, n (%) | 166 (48.97) | | 125 (36.98) | | 126 (37.28) | | 141 (41.59) | | 0.0145 |
| Current smoking, n (%) | 55 (16.22) | | 52 (15.38) | | 52 (15.38) | | 50 (14.75) | | 0.63 |
| Hypertension, n (%) | 122 (35.98) | | 147 (43.49) | | 186 (55.03) | | 220 (64.91) | | <0.001 |
| Diabetes mellitus, n (%) | 50 (14.75) | | 62 (18.34) | | 87 (25.74) | | 110 (32.45) | | <0.001 |
| BMI (kg/m^2^) | 24.93 ± 3.40 | | 25.52 ± 3.69 | | 25.59 ± 3.54 | | 26.09 ± 3.55 | | 0.002 |
| Systolic BP (mm Hg) | 124.79 ± 16.01 | | 128.26 ± 16.83 | | 131.87 ± 17.64 | | 135.17 ± 18.18 | | <0.001 |
| Diastolic BP (mm Hg) | 72.79 ± 8.65 | | 73.12 ± 9.72 | | 74.44 ± 10.39 | | 74.16 ± 11.19 | | 0.139 |
| FBG (mmol/L) | 5.23 ± 1.05 | | 5.39 ± 1.26 | | 5.60 ± 1.42 | | 6.26 ± 2.38 | | <0.001 |
| 2-h PBG (mmol/L) | 7.18 ± 2.65 | | 7.58 ± 2.93 | | 7.82 ± 2.94 | | 9.37 ± 3.53 | | <0.001 |
| TC (mmol/L) | 5.15 ± 1.01 | | 5.13 ± 1.04 | | 5.19 ± 0.92 | | 5.27 ± 1.25 | | 0.327 |
| Triglyceride (mmol/L) | 1.45 ± 0.88 | | 1.49 ± 1.18 | | 1.53 ± 0.83 | | 1.60 ± 0.98 | | 0.299 |
| LDL-C (mmol/L) | 3.15 ± 0.82 | | 3.07 ± 0.84 | | 3.17 ± 0.80 | | 3.25 ± 1.02 | | 0.102 |
| HDL-C (mmol/L) | 1.43 ± 0.36 | | 1.44 ± 0.40 | | 1.41 ± 0.36 | | 1.36 ± 0.36 | | 0.06 |
| Uric acid (μmol/L) | 317.44 ± 76.25 | | 303.70 ± 85.09 | | 298.93 ± 73.26 | | 310.98 ± 85.94 | | 0.03 |
| Homocysteine (μmol/L) | 14.8 (12.2,18.3) | | 14.4 (12.0,17.9) | | 14.5 (12.3,18.7) | | 15.7 (12.7,19.3) | | 0.214 |
| eGFR (ml/min/1.73 m^2^) | 79.98 ± 13.25 | | 81.57 ± 11.94 | | 79.92 ± 11.86 | | 77.64 ± 14.23 | | 0.003 |
| hs-cTnT (pg/mL) | 6.0 (5.0,8.0) | | 6.0 (5.0,9.0) | | 7.0 (5.0,10.0) | | 8.0 (6.0,12.0) | | <0.001 |
| Anti-hypertensive, n (%) | 99 (29.20) | | 111 (32.84) | | 150 (44.38) | | 196 (57.82) | | <0.001 |
| Antidiabetic use, n (%) | 35(10.32) | | 41 (12.13) | | 58（17.16) | | 76 (22.42) | | 0.0004 |
| Lipid lowering use n (%) | 51 (15.04) | | 62 (18.34) | | 57 (16.86) | | 71 (20.94) | | 0.2934 |

Characteristics are reported as counts and percentages for categorical variables and means ± SD or median (with interquartile range) for continuous variables. BMI, body mass index; HDL-C, high density lipoprotein cholesterol; eGFR, estimated glomerular filtration rate; FBG, fasting blood glucose; hs-cTnT, high- sensitivity cardiac troponin T; LDL-C, low density lipoprotein cholesterol; 2-h PBG, 2-h postprandial blood glucose; TC, total cholesterol; UACR, urinary albumin to creatinine ratio.
